# The Association Between Intraoperative Objective Neuromuscular Monitoring and Rocuronium Consumption During Laparoscopic Abdominal Surgery: A Single-Center Retrospective Analysis

**DOI:** 10.7759/cureus.19245

**Published:** 2021-11-04

**Authors:** Kenichi Takahoko, Hajime Iwasaki, Yosuke Inaba, Takashi Matsuno, Risako Matsuno, Sarah K Luthe, Hirotsugu Kanda, Yohei Kawasaki

**Affiliations:** 1 Anesthesiology and Critical Care Medicine, Asahikawa Medical University, Asahikawa, JPN; 2 Clinical Biostatistics, Chiba University Hospital, Chiba, JPN; 3 Anesthesia, Indiana University School of Medicine, Indianapolis, USA; 4 Nursing, Japanese Red Cross College of Nursing, Tokyo, JPN

**Keywords:** elderly patients, laparoscopic surgery, sugammadex, rocuronium, neuromuscular monitoring

## Abstract

Background

Rocuronium consumption with or without intraoperative objective neuromuscular monitoring in clinical settings of unrestricted use of sugammadex and neuromuscular monitoring has not been reported earlier. The study aimed to investigate the association between the use of intraoperative objective neuromuscular monitoring and rocuronium consumption in patients undergoing laparoscopic abdominal surgery.

Methods

Data were collected by reviewing electronic medical records of patients who received laparoscopic abdominal surgery under general anesthesia with rocuronium and reversal with sugammadex at a university teaching hospital between May 2017 and April 2018. A multivariate linear regression model was developed to compare the amount of rocuronium consumption (mg) per weight (kg) per hour (mg/kg/h) between the group in which intraoperative objective neuromuscular monitoring was used (NMM+ group) and the group in which intraoperative neuromuscular monitoring was not used (NMM− group). Additionally, we performed an interaction test.

Results

A total of 429 patients were evaluated, with 371 patients (86%) included in the NMM+ group and 58 patients (14%) in the NMM− group. Log-transformed rocuronium consumption between the NMM+ group and NMM− group was not significantly different (back-transformed *β *coefficients [95% CI]: 1.080 [0.951-1.226]; P = 0.23). Male sex and body mass index (BMI) were independent factors associated with 15% (0.853 [0.788-0.924]; P < 0.001) and 3% (for every 1 kg/m^2^ increase in BMI) (0.971 [0.963-0.979]; P < 0.001) decrease in intraoperative rocuronium consumption, respectively. A significant interaction was detected only between the use of neuromuscular monitoring and age ≥65 years (*β*: 0.803 [0.662-0.974]; P = 0.026).

Conclusions

Although the use of intraoperative objective neuromuscular monitoring was not an individual factor influencing intraoperative rocuronium consumption, this retrospective study demonstrated that the use of intraoperative neuromuscular monitoring reduced rocuronium consumption for approximately 20% of elderly patients (age ≥65 years) undergoing laparoscopic abdominal surgery.

## Introduction

Intraoperative neuromuscular monitoring provides precise measurement of the depth of neuromuscular block as guidance for appropriate dosing of neuromuscular blocking agents (NMBAs) and reversal agents. The use of quantitative (objective) neuromuscular monitoring is recommended whenever NMBAs and neuromuscular reversal agents are administered [1]. We have previously reported that the use of intraoperative neuromuscular monitoring reduces the reversal dose of sugammadex in a single-center retrospective study [2]. To the best of our knowledge, rocuronium consumption with or without intraoperative objective neuromuscular monitoring in clinical settings of unrestricted use of sugammadex and neuromuscular monitoring has not been reported. Since the optimal depth of neuromuscular block differs among the types of surgery, we focused on laparoscopic surgery in which the use of NMBAs is recommended throughout the procedure [3]. In addition, it is reported that elderly patients are at greater risk of postoperative residual neuromuscular block and postoperative pulmonary complications [4]. We hypothesized that the use of intraoperative objective neuromuscular monitoring decreases rocuronium consumption and prevents overuse of rocuronium during laparoscopic surgery in elderly (age ≥65 years) patients. Therefore, the primary aim of this retrospective study was to compare rocuronium consumption in laparoscopic surgical cases in which intraoperative neuromuscular monitor was used to cases in which intraoperative neuromuscular monitoring was not used. Additionally, we analyzed the interaction between the use of intraoperative neuromuscular monitoring and other factors including age on intraoperative rocuronium consumption.

## Materials and methods

A preliminary version of this article was previously posted to the Research Square preprint server on February 4, 2021.

Ethical approval for this retrospective cohort study was provided by the Asahikawa Medical University Research Ethics Committee on March 31, 2020 (approval number: 18242-2). Informed consent was waived due to the retrospective design of the study. This study was conducted at Asahikawa Medical University Hospital in Asahikawa, Japan. Data were collected by using VIPros (Dowell Co., Ltd., Tokyo, Japan) and reviewing the electronic medical records (EMRs) of patients who received laparoscopic abdominal surgery under general anesthesia maintained with rocuronium and reversed with sugammadex at our hospital between May 1, 2017 and April 30, 2018. Patients with incomplete medical records were excluded. The collected data included the use of intraoperative neuromuscular monitoring (IntelliVue NMT Module, Philips Healthcare, Amsterdam, The Netherlands), characteristics of patient, surgery, and anesthesia.

Patient characteristics included age, sex, body mass index (BMI [kg/m^2^]), liver damage marker enzyme of alanine aminotransferase (ALT [IU/L]), and renal function as estimated glomerular filtration rate (eGFR [ml/min/1.73 m^2^]). Surgical characteristics included type of surgery and surgical procedure time (minutes). Anesthesia characteristics included anesthesia time (minutes), type of anesthetic agent (inhalational or intravenous), the total dose of administered rocuronium (mg), and type of additional rocuronium administration (continuous infusion or intermittent bolus).

The main outcome in this study was the difference in the total amount of rocuronium consumption (mg) per weight (kg) per surgical duration (hours) (mg/kg/h) between the group in which intraoperative quantitative neuromuscular monitoring was used (NMM+ group) and the group in which intraoperative neuromuscular monitoring was not used (NMM− group). In addition, we performed an interaction test.

Statistical analysis

Data are shown as percentage (number), median (25-75%, interquartile range [IQR]), or mean difference (95% confidence interval [CI]). For univariate comparisons, we used the χ2 test for categorical variables and Student’s t-test or Mann-Whitney U test for continuous variables.

Multivariate linear regression analysis was performed to compare the amount of intraoperative rocuronium consumption (mg/kg/h) between the NMM+ group and NMM− group after adjusting for confounding factors. The primary independent variable was the use of intraoperative neuromuscular monitoring. Other independent variables were possible confounding factors previously reported to influence neuromuscular blocking effects: age [5], sex [6], BMI [7], liver function (we used liver damage marker enzyme of ALT) [8], renal function (eGFR) [9], type of anesthetic agent [10], and type of maintenance rocuronium administration (bolus vs. continuous infusion) [11]. A subject-to-variable ratio of 15:1 was maintained to avoid overfitting of independent variables. The dependent variable was the amount of intraoperative rocuronium consumption (mg/kg/h). All variables were decided prior to the analysis based on the aforementioned report and forced into the model. The assumptions of linearity, normality, homoscedasticity, and multicollinearity were tested using two-way scatter plots, normal quantile-quantile (QQ) plots, residuals vs. fitted plots, and variance inflation factors. Adjusted R-squared was calculated to measure the model’s goodness of fit. Potential effect variables including age (<65 or ≥65 years), sex (male or female), BMI (<18.5 or 18.5-25 or 25≤), ALT (<40 or ≥40), eGFR (<60 or ≥60), and type of anesthetic agent (inhalational or intravenous) were tested for interaction with the use of neuromuscular monitoring in the final multivariate linear regression analysis.

Three propensity score (PS) analyses were performed as a sensitivity analysis to control for confounding factors between the NMM+ group and NMM− group. All confounding factors used in the multivariate linear regression analysis were included in the PS model. Differences in covariates between the NMM+ group and NMM− group were assessed using the standardized difference. First, we conduct a multivariate linear regression with covariates of PS, age, the use of intraoperative neuromuscular monitoring, as well as the interaction between age and the use of intraoperative neuromuscular monitoring. Second, the inverse probability weighting (IPW) method was performed to adjust for the PS score difference. Lastly, PS matching was conducted using nearest neighbor matching without replacement. A caliper was fixed at 20% of the standard deviation (SD) of the logit of the PSs.

A two-sided significance level of 5% was used in all statistical analyses. All analyses were performed by R software, version 3.4.1 (R Foundation for Statistical Computing, Vienna, Austria) and SAS, version 9.4 (SAS Institute, Cary, NC, USA).

## Results

Among 459 patients who received laparoscopic abdominal surgery under general anesthesia maintained with rocuronium and reversed with sugammadex from May 2017 to April 2018, we excluded 30 patients who had incomplete medical records. A total of 429 patients were evaluated, with 371 patients (86%) included in the NMM+ group and 58 patients (14%) in the NMM− group. Patient characteristics are shown in Table 1. There were no significant differences in age, sex, BMI, ALT, eGFR, type of surgery, and type of anesthetic agent between the two groups. NMM+ group patients had longer surgery and anesthesia time and were more likely to receive additional rocuronium by intermittent bolus than NMM− group patients. In the NMM+ group, 94% of the patients received additional rocuronium after the reappearance of train-of-four (TOF) response while 6% of the patients received additional rocuronium before the reappearance of TOF to maintain post-tetanic count (PTC). Reversal dose of sugammadex was decided based on the response of neuromuscular monitoring in the NMM+ group and according to the attending anesthesiologist’s subjective evaluation in NMM− group. NMM− group patients received significantly larger reversal doses of sugammadex compared to the NMM+ group (2.2 [2.1-2.9] vs. 2.1 [2.0-2.5]).

**Table 1:** Patient characteristics. Data are expressed as % (n) or median (25-75%, interquartile range [IQR]). Abbreviations: NMM+, with intraoperative objective neuromuscular monitoring; NMM−, without neuromuscular monitoring; BMI, body mass index; ALT, alanine aminotransferase; eGFR, estimated glomerular filtration rate.

	Group NMM+ (n = 371)	Group NMM− (n = 58)	P-value
Age (years)	65 (49-72)	63 (46-72)	0.45
Male sex	54.2% (201)	41.4% (24)	0.26
BMI (kg/m^2^)	22.7 (20.6-25.6)	22.2 (19.9-25.4)	0.40
ALT (U/L)	17 (12-24)	17 (14-23)	0.80
eGFR (mL/min/1.73 m^2^)	78 (64-93)	81 (67-99)	0.178
Type of surgery			0.24
Gastroenterology	56.6% (210)	44.8% (26)	
Gynecology	20.8% (77)	27.6% (16)	
Urology	22.6% (84)	27.6% (16)	
Surgical procedure time (min)	202 (128-296)	138 (110-246)	0.019
Anesthesia time (min)	279 (195-374)	215 (173-313)	0.007
Anesthetic agent			0.26
Inhalational	63.9% (237)	55.2% (32)	
Intravenous	36.1% (134)	44.8% (26)	
Additional rocuronium administration			0.013
Intermittent bolus administration	97.3% (361)	89.7% (52)	
Continuous infusion	2.7% (10)	10.3% (6)	
Dose of sugammadex (mg/kg)	2.1 (2.0-2.5)	2.2 (2.1-2.9)	0.010

Table 2 illustrates the multivariate linear regression model developed to compare intraoperative rocuronium consumption (mg/kg/h) between the groups. Dependent variables were logarithmically transformed since the distribution of the residuals was positively skewed in the normal QQ plot, thereby generating a normal distribution. The resulting *β* coefficients from the log-transformed outcome were back-transformed. The back-transformed *β* coefficients were then interpreted as a percentage increase or decrease. The F-value and P-value of the model were 22.09 and <0.001, respectively, indicating that the model was accurate. The model’s adjusted R-squared was 0.31, indicating that 31% of the variance could be explained by the model. Rocuronium consumption between NMM+ group and NMM− group was not significantly different (*β* coefficients [95% CI]: 1.054 [0.946-1.174]; P = 0.34). Male sex was independently associated with decreased rocuronium consumption by approximately 15% (*β*: 0.853 [0.788-0.924]; P < 0.001). BMI was also an independent factor associated with a 3% decrease in rocuronium consumption for every 1 kg/m^2^ increase in BMI (*β*: 0.971 [0.963-0.979]; P < 0.001). Rocuronium consumption tended to increase with age <65 years, decrease in ALT, increase in eGFR, intravenous anesthesia (vs. inhalational), and bolus administration of rocuronium (vs. continuous infusion), although statistical significance was not achieved.

**Table 2:** Multivariate linear regression model developed for estimating the dose of rocuronium consumption (mg) per weight (kg) per hour (mg/kg/h). Adjusted R-squared: 0.31. F-value: 22.09, P-value: <0.001. *β* coefficients and 95% confidence intervals are back-transformed coefficients from multivariate linear regression models using log-transformed dependent variables. Abbreviations: CI, confidence interval; BMI, body mass index; ALT, alanine aminotransferase; eGFR, estimated glomerular filtration rate.

Variables	*β* coefficients (95% CI)	P-value
Use of intraoperative neuromuscular monitoring	1.080 (0.951-1.226)	0.23
Age ≥65 years	0.986 (­0.821-1.185)	0.88
Male sex	0.853 (0.788­-0.924)	<0.001>
BMI	0.971 (0.963­-0.979)	<0.001>
ALT	1.000 (0.999­-1.000)	0.25
eGFR	1.001 (1.000­-1.002)	0.138
Intravenous anesthesia (vs. inhalational)	1.068 (0.978­-1.166)	0.145
Bolus administration (vs. continuous)	1.047 (0.878­-1.249)	0.61
Interaction		
Use of intraoperative neuromuscular monitoring and age ≥65 years	0.803 (0.662­-0.974)	0.026

A significant interaction was detected between the use of neuromuscular monitoring and age ≥65 years (*β*: 0.803 [0.662-0.974]; P = 0.026) (Table 2).

For the first sensitivity analysis, we used multivariate linear regression with PS score as a covariate, in which a significant interaction was detected between age ≥65 years and the use of intraoperative neuromuscular monitoring (*β*: 0.802 [0.650-0.989]; P = 0.038). For the second sensitivity analysis, we used the IPW method, in which a significant interaction was revealed between age ≥65 years and the use of intraoperative neuromuscular monitoring (*β*: 0.764 [0.665-0.878]; P < 0.001). For the final sensitivity analysis, we used PS matching, in which the interaction between age ≥65 years and the use of intraoperative neuromuscular monitoring was not significant, however, the P-value was close to the threshold (*β*: 0.786 [0.600-1.032]; P = 0.083).

## Discussion

In this single-center retrospective study, although the use of intraoperative neuromuscular monitoring was not an individual factor influencing intraoperative rocuronium consumption, we found that the use of intraoperative neuromuscular monitoring reduced rocuronium consumption by approximately 20% during laparoscopic surgery in elderly patients (age ≥65 years). In other words, without intraoperative neuromuscular monitoring, there is a risk of overusing rocuronium in elderly patients. It is known that elderly patients have a longer duration of action of rocuronium compared to younger patients [5,12]. One prospective study showed that mean ± SD of the duration of action (minutes) after 0.6 mg/kg rocuronium administration in elderly and younger controls were 42.4 ± 14.5 and 27.5 ± 7.1, respectively [5]. Moreover, elderly patients have greater variability in the duration of action of rocuronium compared to younger patients [13]. One prospective study reported that the range of duration of action after 0.6 mg/kg rocuronium administration under sevoflurane anesthesia in elderly patients was 33-119 minutes [13]. The mechanism of this prolongation and increased variability in the duration of action of rocuronium in elderly patients are attributed to decreased plasma clearance of rocuronium due to age-related reduction in liver size [14], decrease in hepatic and renal blood flow [14], and decrease in rocuronium’s distribution volume [5]. The lower rocuronium consumption in elderly patients who underwent quantitative monitoring vs. those who did not undergo such monitoring (and were managed with subjective evaluation) indicates that quantitative monitoring facilitates the administration of lower total doses of rocuronium. This may indicate patient safety implications since the elderly are at higher risk for residual neuromuscular block and attendant complications [4].

According to previous reports, age [5], female sex [6], obesity [7], liver dysfunction [8], renal dysfunction [9], use of inhalational anesthesia [10], and continuous infusion of rocuronium [11] are the factors which decrease intraoperative rocuronium consumption. In our study’s multivariate linear regression analysis, BMI and male sex were the independent factors associated with a significant decrease in intraoperative rocuronium consumption per body weight. Therefore, factors other than male sex, which decreased rocuronium consumption in our study, were consistent with previous reports. One prospective study showed that women required approximately 30% less rocuronium than men to maintain the same degree of neuromuscular block [6]. The etiology of the high sensitivity to NMBAs in the female sex was reported as more adipose tissue, less muscle, and lower volume of distribution compared to men [6]. In contrast, the male sex had a decrease in rocuronium consumption by 15% in our study. The reason for the apparent contradiction is unknown; however, we speculate that a higher age group (median age of approximately 65 years old) in our study compared to a previous study (mean age of approximately 30 years old) may have affected the different sensitivity to NMBAs between sex.

Consistent with our previous report [2], we found that the use of intraoperative neuromuscular monitoring significantly reduced the reversal dose of sugammadex in patients undergoing laparoscopic abdominal surgery. In our previous study [2], we compared the reversal dose of sugammadex between NMM+ and NMM− regardless of the type of surgery. We believe that the findings of the present analysis of laparoscopic abdominal surgical patients support our previous conclusion that the use of intraoperative neuromuscular monitoring may reduce the reversal dose of sugammadex.

During laparoscopic surgery, it is recommended to maintain a deeper neuromuscular block to improve the quality of the surgical field and to reduce the risk of intraoperative adverse events [15]. However, a remaining question is, how deep should the neuromuscular block be during laparoscopic surgery? A recent systematic review concluded that the use of deep neuromuscular block (defined as PTC 1-2) [1] may improve laparoscopic surgical conditions compared with the moderate neuromuscular block (TOF count 1-2) [16]. Moreover, a randomized double-blind controlled study reported that deep neuromuscular block improved not only surgical conditions but also postoperative pain in laparoscopic bariatric surgery [17]. In the NMM+ group in our study, 94% of the patients were maintained with a moderate neuromuscular block during the surgery. As the first limitation of this study, if all patients were maintained with a deep neuromuscular block in the NMM+ group in our study, the difference in intraoperative rocuronium consumption between the NMM+ group and NMM− group may have been less.

This study has several other limitations. First, as our study was a single-center study, our data may not be applicable to other institutions. However, the present results will be an example of an institution in which routine practice includes unrestricted use of sugammadex and the widespread availability of quantitative neuromuscular monitors in every operating room. Second, in NMM− group, additional rocuronium was administered according to the attending anesthesiologist’s subjective evaluation. Subjective evaluation is likely based on each individual’s clinical experience, which can be affected by institutional factors and educational background. Given that there was no statistical difference in rocuronium consumption between the NMM+ group and NMM− group among young patients (age <65 years), we anticipate that excessively high or low doses of rocuronium were not administered even without the use of intraoperative neuromuscular monitoring in our study. Third, due to the relatively high usage (86%) of intraoperative neuromuscular monitoring in this study, the number of patients in the NMM− group was small. Finally, as with any observational study, the observed associations may be confounded by unmeasured factors such as patient severity. However, the association of interest remained significant after accounting for previously reported confounding factors.

## Conclusions

In conclusion, this single-center retrospective study demonstrated that although the use of intraoperative objective neuromuscular monitoring was not an individual factor influencing intraoperative rocuronium consumption, the use of intraoperative neuromuscular monitoring reduced rocuronium consumption by approximately 20% during laparoscopic abdominal surgery in elderly patients (age ≥65 years). Male sex and BMI were independent factors associated with decreased rocuronium consumption by 15% and 3% (for every 1 kg/m^2^ increase in BMI), respectively. Rocuronium consumption tended to increase with age <65 years, decrease in ALT, increase in eGFR, intravenous anesthesia (vs. inhalational), and bolus administration of rocuronium (vs. continuous infusion). Our findings should facilitate further research into the potential role of objective intraoperative neuromuscular monitoring in maintaining the appropriate depth of neuromuscular block and optimization of NMBAs as well as antagonist consumption to prevent their overuse.
